# RENATE: A Pseudo‐retrosynthetic Tool for Synthetically Accessible *de novo* Design

**DOI:** 10.1002/minf.202100207

**Published:** 2021-11-08

**Authors:** Gian Marco Ghiandoni, Michael J. Bodkin, Beining Chen, Dimitar Hristozov, James E. A. Wallace, James Webster, Valerie J. Gillet

**Affiliations:** ^1^ Information School University of Sheffield Regent Court, 211 Portobello Sheffield S1 4DP UK; ^2^ Evotec (U.K.) Ltd 114 Innovation Drive Milton Park Abingdon OX14 4RZ UK; ^3^ Chemistry Department University of Sheffield Dainton Building, Brook Hill Sheffield S3 7HF UK

**Keywords:** *de novo* drug design, reaction informatics, patents, pharmaceuticals

## Abstract

Reaction‐based *de novo* design refers to the generation of synthetically accessible molecules using transformation rules extracted from known reactions in the literature. In this context, we have previously described the extraction of reaction vectors from a reactions database and their coupling with a structure generation algorithm for the generation of novel molecules from a starting material. An issue when designing molecules from a starting material is the combinatorial explosion of possible product molecules that can be generated, especially for multistep syntheses. Here, we present the development of RENATE, a reaction‐based *de novo* design tool, which is based on a pseudo‐retrosynthetic fragmentation of a reference ligand and an inside‐out approach to *de novo* design. The reference ligand is fragmented; each fragment is used to search for similar fragments as building blocks; the building blocks are combined into products using reaction vectors; and a synthetic route is suggested for each product molecule. The RENATE methodology is presented followed by a retrospective validation to recreate a set of approved drugs. Results show that RENATE can generate very similar or even identical structures to the corresponding input drugs, hence validating the fragmentation, search, and design heuristics implemented in the tool.

## Graphical abstract

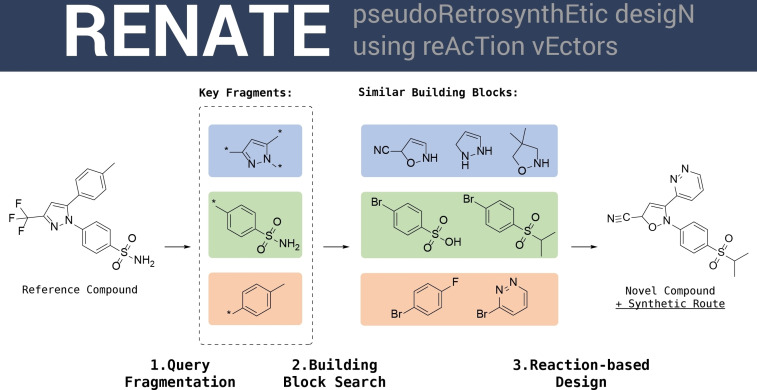

## 1 Introduction

*In‐silico de novo* drug design consists of creating novel molecules with desired properties.^[1]^ These properties include biological activity, physicochemical properties, pharmacokinetics, toxicity, and synthetic accessibility. Although first introduced more than 30 years ago,^[2]^ *de novo* design remains a very challenging task due to the complex relationships amongst these properties and the astronomical number of valid structures that could be generated. A major limitation of early *de novo* design was the inability to account for the synthetic accessibility of products, which resulted in its poor application to real problems.^[3]^ Reaction‐based methods were introduced with the aim of accounting for this limitation. These used small numbers of hard‐coded transformation rules (e. g. organic chemistry reactions)[^4^, ^5^] to address synthetic accessibility explicitly. More recently AI‐based methods have gained considerable popularity for *de novo* design.[^6^, ^7^] The initial focus was on the adoption of techniques developed in natural language processing to generate molecules as SMILES representations, with methods then extended to graph representations. However, there is no explicit handling of synthetic accessibility in these approaches.^[8]^ Attention has now turned towards combining reaction‐based methods with machine learning techniques for molecular generation. For example, machine learning has been used to select building blocks to combine using reaction rules^[9]^ and to select preferred reactions to apply to a given starting material.^[10]^ Reaction‐based methods are now also being integrated with deep reinforcement learning methods[^11^, ^12^] where the reinforcement learning is used to select which reaction template to apply next. A more complex deep learning approach has recently been proposed based on synthesis directed acyclic graphs (synthesis DAGs) which encodes synthetic routes as reaction schemes representations.^[13]^

The availability of large collections of publicly available reactions has enabled reaction‐based *de novo* design to be extended beyond a small set of hand coded reaction rules. We have adapted reaction vectors for this purpose.^[14]^ The reaction vector encodes the structural changes that take place during a reaction into a vector with negative and positive counts of atom pair descriptors indicating fragments that are removed from the reactants and introduced into the products, respectively. Reaction vectors can be calculated by simply subtracting the vector that represents the reactants from that representing the products. We have coupled the reaction vector with a structure generation procedure which allows a reaction vector to be applied to a new starting material and, provided that the starting material contains the fragments to be removed (that is, atom pairs corresponding to the negative atom pairs of the reaction vector), a new product can be generated. The reaction vectors are comprised of atom pair 2 and atom pair 3 descriptors with atom pair 2 descriptors encoding the bond changes in a reaction and atom pair 3 descriptors extending the environment of the reaction that is encoded in a sphere‐based manner.

We have shown that reaction vectors can encode chemistry correctly by applying them to their original reactants to reproduce their corresponding products.^[15]^ We have also demonstrated the use of reaction vectors for data‐driven reaction classification by developing a model called SHREC with a set of reactions extracted from the US patent literature.^[16]^ In a recent publication, we build a reaction class recommender to enhance the synthetic accessibility of reaction vector products by predicting which reactions to apply to an input molecule according to its fingerprint.^[10]^ In the same publication, we briefly described the validation of the recommender using an automated *de novo* drug design tool called RENATE (REtrosynthetic desigN using reAcTion vEctors).

An issue when designing molecules from a small starting material is the huge combinatorial explosion of possible product molecules that can be generated especially when multistep syntheses are considered. RENATE aims to circumvent the combinatorial explosion by taking an inside‐out approach to *de novo* design. It is based on the principles of pseudo‐retrosynthetic *de novo* design which was first proposed over 10 years ago.[^17^, ^18^] For example, in the Flux program a molecule is fragmented using a small set of retrosynthetic rules. The same rules are used to fragment molecules in a database which then form building blocks for *de novo* design. The fragments of the target molecule are used to identify similar building blocks which are then recombined to generate novel molecules. The term *pseudo* refers to the simplistic retrosynthetic approach that breaks the bonds in a molecule without accounting for functional conversions, eliminations, or ring transformations. In Flux, the rules are the 11 bond‐cleavage types implemented in RECAP^[19]^ and the building blocks are connected via attachment points determined using RECAP. The key advance in RENATE is in the construction phase where the retrieved fragments are combined using reaction vectors. The product molecules are therefore based on reactions for which precedents exist in the literature and synthetic accessibility is accounted for explicitly by the use of real reagents and reactions during the structure generation step.

Here we describe the implementation of RENATE in detail and its retrospective validation on a large set of approved drugs. We show that RENATE can be controlled by user‐defined sources of reagents and reactions, scoring functions and parameters. We compare the use of two reaction vector sources from the US patent literature and the Journal of Medicinal Chemistry, respectively, and determine an optimal setup for *de novo* design. The results from the validation demonstrate that RENATE can generate relevant drug candidates and provide references for their synthesis. The use of RENATE in a real case study will be described in a future publication.

## 2 Methods

### 2.1 The RENATE Algorithm

**Figure 1:**
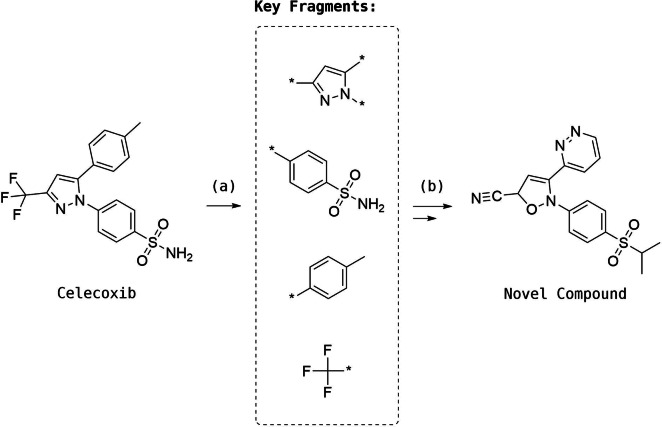
Pseudo‐retrosynthetic *de novo* design applied to the molecule Celecoxib. The query ligand bonds are (a) broken to yield a set of key fragments. Fragments are then used to retrieve similar structures that are (b) recombined to yield novel compounds that are similar to the query ligand.

RENATE is a pseudo‐retrosynthetic *de novo* design tool that fragments a molecule, searches for sets of similar fragments and then combines these using the reaction‐based structure generator to generate novel product molecules. Synthetic accessibility is accounted for explicitly through the use of reaction vectors which are derived from known reactions and fragments which are extracted from a database of available molecules. RENATE is composed of four modules: *ligand fragmentation*, *building block search*, *structure generation* and *scoring*. An example of a pseudo‐retrosynthetic *de novo* design scheme is illustrated in Figure 1.

**Figure 2:**
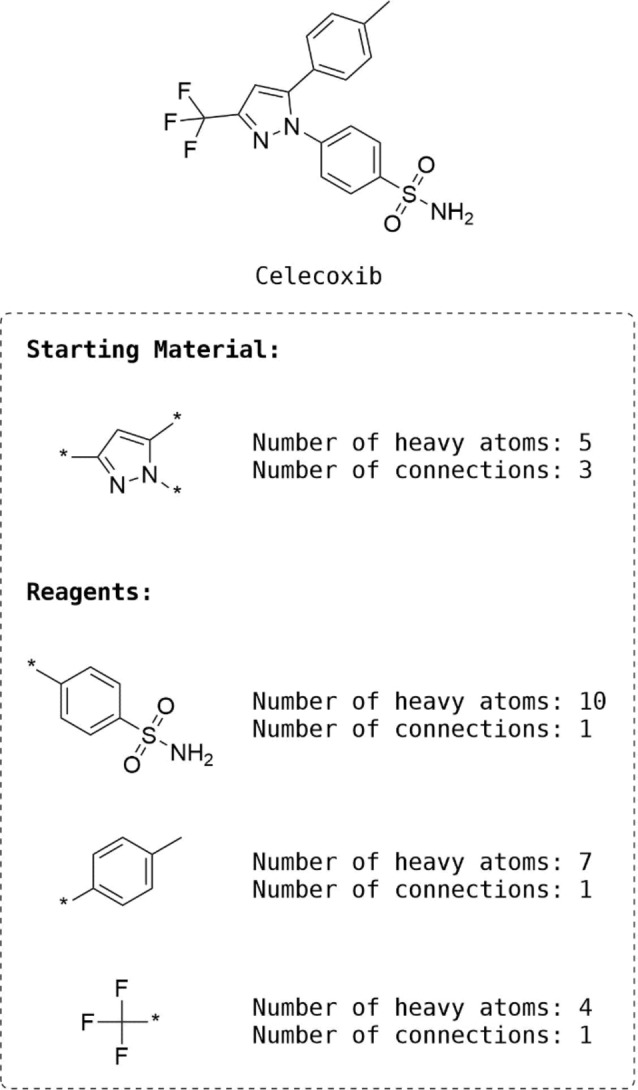
Ligand fragmentation results for the molecule Celecoxib. The pyrazole is identified as the starting material (molecular scaffold) due to the higher number of connections compared to the other fragments, which are, therefore, considered as reagents (substituents).

The *ligand fragmentation* module breaks a reference ligand or query into key fragments from which the molecular scaffold is identified with the remaining fragments identified as substituents. The fragmentation is done using the BRICS^[20]^ module in RDKit. BRICS can be considered an extension of the RECAP approach that consists of 16 bond‐cleavage rules. Once key fragments are produced, they are sorted first by descending number of connections and then by number of heavy atoms. The fragment at the top of the ranked list is identified as the scaffold and forms the basis of the search for starting materials. This heuristic ensures that the candidate molecules are constructed from ‘the inside out’. The fragmentation module is controlled by two parameters: *minFragmentSize* and *minKeyFragSize. minFragmentSize* determines which bonds can be broken in BRICS according to the resulting fragment size, whereas, *minKeyFragSize* is the sum of the number of heavy atoms and the number of connections and is used to filter out fragments that are too small to be considered relevant, hence reducing the number of design iterations. An example of the fragmentation procedure is shown in Figure 2 for Celecoxib.

The *building block search* module takes the scaffold and each substituent fragment in turn and performs a search on an external source of structures (e. g. a reagent catalogue) to retrieve similar reagents based on a user‐defined similarity measure. In the examples shown here similarity is calculated using 1024‐bit binary Morgan fingerprints (radius 2) and the Tanimoto coefficient. For each fragment query, the search returns a set of starting materials and sets of reagents sorted on similarity to the parent fragment. For example, given a scaffold ‘A’ and a substituent ‘B’, the algorithm returns two sets of reagents (e. g. {a_1_, a_2_, …, a_x_}, {b_1_, b_2_, …, b_y_}) scored by similarity. The sizes of the sets are controlled by the parameters *maxStartingMaterials* and *maxReagents*.

The *structure generation* module implements a logic similar to that in synthetic chemistry. In the first design cycle, the structure generator combines the fragments retrieved for the scaffold (considered as starting materials) with those retrieved for the first substituent (considered as reagents) using the available set of reaction vectors. For example, the sets {*a*_1_, *a*_2_, …, *a_x_*} and {*b*_1_, *b*_2_„ …, *b_y_*} are considered. For each pair of fragments (one from set *a* and one from set *b*), the set of reaction vectors is searched and for each applicable reaction vector, a product molecule is generated. A reaction vector is applicable if its negative atom pairs, which encode the parts of reactant(s) that are transformed into product, are present within the combined atom pairs of the starting material and reagent currently being processed. Not all reaction vectors will be applicable to all pairs of fragments. This step results in a set of product molecules of the form (*a*_1_‐*b*_3_, *b*_2_‐*a*_5_, …, *a*_9_‐*b*_11,…_}. The products are scored (see below), and the top scoring products form a new set of starting materials. These are then input to the structure generator to be combined with the reagents retrieved for the second substituent (e. g. {c_1_, c_2_, …, c_z_}). The algorithm iterates until all substituents have been considered. At the end of each iteration, the product selection is controlled using two more parameters: *queryHeavyAtomsAddThreshold* is used to filter out products according to the size of the query ligand. For example, if q*ueryHeavyAtomsAddThreshold* is equal to 0.25, then products exceeding 25 % of the heavy atom count of the query ligand are filtered out. *numProductsCycle* determines how many products are retained at the end of each iteration for the next round of structure generation.

**Figure 3:**
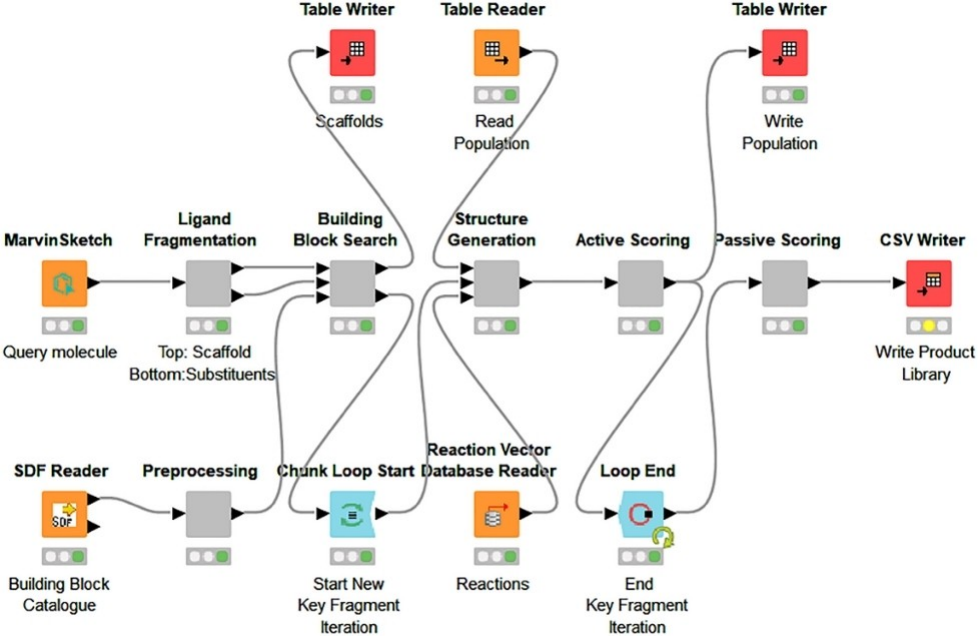
RENATE KNIME workflow: Query molecules are fragmented and used to find fragments similar to the scaffold (which form starting materials) and each of the substituents (to form lists of reagents). The fragments returned from the scaffold search are written to a temporary file, which is read by the structure generator as a starting population. Once the starting materials have been combined with the first set of reagents, the new population is scored and overwrites the temporary table. RENATE iterates through each reagent set while reading and overwriting the temporary table until the process is complete. The final population is then rescored and written out.

The *scoring* module is defined by the user. In the simplest implementation of RENATE, it is configured as a similarity‐based method that selects the best molecules based upon their similarity to the query. The *scoring module* first drives the design by selecting the best products at the end of each cycle (*active* scoring), then finally sorts the entire population of intermediates and final products to yield a set of candidates (*passive* scoring). The total number of candidates retained by the algorithm for a given query is controlled by the parameter *numFinalProducts*. A GUI version of RENATE was implemented using KNIME^[21]^ as described in Figure 3.

**Figure 4:**
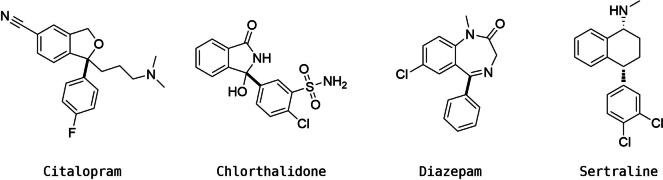
Drugs that failed the BRICS decomposition. Potential fragmentation bonds are highlighted in bold.

## 3 Retrospective Validation

RENATE was validated by applying it to a set of approved drugs to assess whether the algorithm could either recreate the drugs or molecules that are similar to the drugs. The experiment had two aims: first, to verify the assumptions made by the algorithm (correct fragmentation and starting material/reagent role assignment) and, second, to determine whether the selected reagent catalogue and reaction vector databases would enable effective designs in drug‐like space.

A collection of drugs from the top 200 medicines prescribed in the US in the year 2017 (https://clincalc.com/DrugStats/) was used as a reference set for the validation. The drugs were drawn using MarvinSketch and converted into SMILES which were sanitised using RDKit,^[22]^ then salts and ions were stripped to obtain one molecule per entry. Several filters were applied to obtain a subset of the drugs to benchmark the performance of RENATE: a minimum of 20 atoms (heavy atoms and hydrogens), a maximum of three fused rings, a minimum of two rings, a maximum of one Lipinski's violation.^[23]^ Next, a diverse subset was selected by calculating the pairwise similarities between all molecules and retaining only one molecule for each pair with a similarity greater than or equal to 0.6. Similarities were calculated using 1024‐bit binary Morgan fingerprints (radius 2) and the Tanimoto coefficient. The filtering yielded 73 structures which are reported in the Supporting Information.

A set of 746,272 reagents was obtained from the Enamine website (sanitised using RDKit, neutralised, then deduplicated using InChI Keys) and selected as a source of starting materials and reagents. Any of the 73 reference drugs that were present in the Enamine set were filtered out to prevent the algorithm from picking the complete drug structure during the design. Two different sources of reaction vectors were used: 92,530 obtained from the US Patent Database (referred to as USPD); and 7,109 obtained from the Journal of Medicinal Chemistry (JMC 2018). The USPD reaction vectors were obtained following the encoding of the 115 K unique reactions as described in Ghiandoni et al.^[16]^ The JMC 2018 set consisted of 7,109 unique reaction vectors obtained from 27 K reactions as described in Ghiandoni et al.^[10]^ 1024‐count Morgan fingerprints (radius 2) and Euclidean distance were selected for the scoring of both building blocks and products. The selection of structural fingerprints as scoring function was aimed at maximising the chance of reproducing the reference drugs.

The design workflows were carried out using the USPD and JMC 2018 reaction vectors, respectively, in order to determine the sensitivity of the approach to the source of reaction vectors. The parameters used in the experiments are reported in the Supporting Information.

## 4 Results

11 of the drugs (15 %) failed the BRICS fragmentation step. The decomposition mainly failed due to the lack of rules for the fragmentation of single bonds between aromatic and aliphatic rings. These failures indicate that BRICS lacks some important fragmentation rules. Some examples of failed queries are reported in Figure 4. The remaining 62 drugs were successfully processed by both BRICS and RENATE.

The top scoring compound (closest reproduction) per drug was retained and the pairwise similarity to the parent drug was calculated using four binary fingerprints: 1024‐bit RDKit Morgan (radius 2) (equivalent of ECFP4) and CDK ECFP4, 1024‐bit RDKit FeatMorgan (radius 2) (equivalent of FCFP4) and CDK FCFP4. The minimum, maximum, mean and median pairwise similarities for the 62 drugs are reported in Table 1 for the USPD and JMC 2018 designs.

**Table 1:** Statistics from the pairwise similarities between queries and their closest reproductions from the USPD and JMC 2018 designs.

Design	Binary Fingerprint	Min	Max	Mean	Median
USPD	RDKit‐ECFP4	0.19	1.00	0.62	0.60
	CDK‐ECFP4	0.18	1.00	0.62	0.61
	RDKit‐FCFP4	0.29	1.00	0.64	0.64
	CDK‐FCFP4	0.23	1.00	0.65	0.64
JMC 2018	RDKit‐ECFP4	0.15	1.00	0.51	0.48
	CDK‐ECFP4	0.12	1.00	0.50	0.48
	RDKit‐FCFP4	0.16	1.00	0.51	0.45
	CDK‐FCFP4	0.21	1.00	0.52	0.52

**Figure 5:**
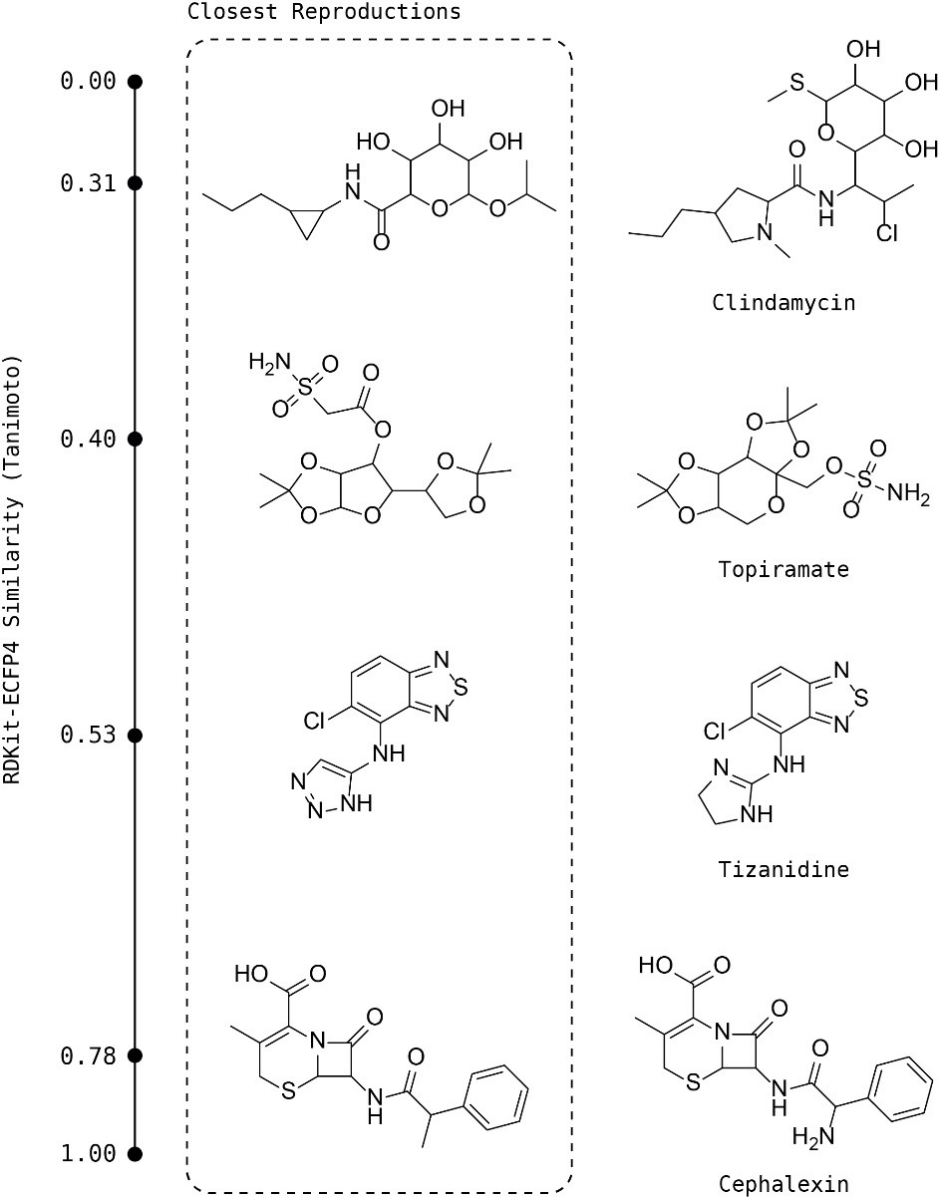
Examples of some closest reproduction‐drug pairwise similarities using RDKit‐ECFP4 generated from the USPD design.

The USPD and JMC 2018 values in Table 1 show that the USPD reaction vectors produced structures that were on average more similar to the target drugs. This is not surprising as the USPD database contains 13 times the number of reaction vectors as the JMC 2018 database. Each top‐scoring compound from the USPD design was also visually inspected and compared with the parent drug. Some examples of the top candidates and their parent drugs are reported in Figure 5, sorted on increasing similarity. This shows that candidates with similarities greater than 0.5 are very similar to the parent drug. For example, the top scoring candidate for Tizanidine (0.53 similarity) presents minor variations on the five‐membered ring, and Cephalexin's candidate (0.78 similarity) differs only in the substitution of an amino group with a methyl. 70 % and 47 % of the top scoring candidates had similarity greater than 0.5 to the parent for the USPD and JMC 2018 experiments, respectively. These results suggest that the USPD reactions is a preferred source of reaction vectors for *de novo* design compared to the considerably smaller JMC 2018 set.

The USPD and JMC 2018 pipelines reproduced 6 and 1 of the drugs, respectively. More specifically, using the USPD reaction vectors, three, two, and one of the drug were regenerated via 1‐step, 2‐step, and 3‐step synthetic routes, respectively, while for the JMC 2018 reaction vectors the one drug that was reproduced was using a 2‐step synthetic route. The virtual synthetic routes of the reproduced drugs were also inspected and compared with the actual routes used to produce the drugs using the original patents as references. Table 2 compares the number of virtual and real synthetic steps (using the original patents as references) per reproduced drug. The comparison between virtual and the real synthetic schemes in the patents revealed that none of the drugs was reproduced using their original references. This result can be rationalized as follows. First, some of the reference patents were not issued in the US, hence their reactions are not necessarily in the USPD set. Second, patents often describe combinations of small and cheap building blocks, whereas RENATE makes use of a vast catalogue of reagents, which can also contain analogues of the queries (e. g., the Naproxen and Levofloxacin designs represent cases of 1‐step conversion of drug analogues into their queries). Third, the use of pseudo‐retrosynthesis does not decompose ligands into their actual precursors (e. g., the synthesis of Rivaroxaban contains a ring closure step that cannot be reversed by fragmentation). Fourth, real syntheses often involve protection chemistry, which is not treated explicitly by reaction vectors.^[10]^

**Table 2:** Virtual and real synthetic steps, plus original patent references, for each reproduced drug from the USPD and JMC 2018 designs.

Design	Drug	Virtual Steps	Real Steps (Patent Reference)
USPD	Brimonidine	1	3 (US3890319 A)
	Glipizide	2	2 (DE2012138)
	Glyburide	2	3 (DE1283837)
	Levofloxacin	1	7 (US4382892 A)
	Naproxen	1	8 (US3896157)
	Rivaroxaban	3	4 (US7157456B2)
JMC 2018	Diclofenac	2	4 (DE1793592)

## 5 Conclusions

We have implemented a pseudo‐retrosynthetic *de novo* design tool referred to as RENATE that incorporates our reaction‐based structure generator. We have reported a retrospective validation of the tool using a set of top prescribed drugs to verify the assumptions on which it relies and to determine an optimal configuration for real *de novo* design. We have shown that the algorithm can explore (i. e., direct the search towards the region of chemical space occupied by the known drug) and also exploit (i. e., reproduce the reference drugs or at least generate very similar candidates) chemical space effectively when sufficient amounts of building blocks and reaction vectors are provided. The algorithm has also been demonstrated to be able to performs valid fragmentations, retrieve relevant reagents, and combine them logically. The validation presented here is based on the retrospective recreation of known drugs with the scoring functions being based on similarity to the target compound. While this strategy presents a useful validation of the methods the real test of the method would be the prospective design of previously unknown compounds based on scoring functions such as QSAR or docking. Such a prospective design will be described in a future publication.

## Statement of Contribution

G.M.G. implementated the approach. All authors contributed to the design of the study and the writing of the manuscript.

## Supporting Information

The following is provided as supporting information:

- 73 structures used in the validation of RENATE that were retained from the top 200 prescribed drugs the US in 2017.
- The parameters used to control RENATE in the USPD and JMC 2018 designs.

## Conflict of interest

None declared.

6

## Supporting information

As a service to our authors and readers, this journal provides supporting information supplied by the authors. Such materials are peer reviewed and may be re‐organized for online delivery, but are not copy‐edited or typeset. Technical support issues arising from supporting information (other than missing files) should be addressed to the authors.

Supporting Information

## Data Availability

Data subject to third party restrictions
