# Supplementary material for: RENATE: A Pseudo‐retrosynthetic Tool for Synthetically Accessible de novo Design
Source: Mol Inform. 2021 Nov 8;41(4):2100207. doi: 10.1002/minf.202100207 (PMC9285524; doi:10.1002/minf.202100207)
Supplement: Supplementary file 1 — Supporting Information [file MINF-41-0-s001.pdf]

molecular informatics

Supporting Information

RENATE: A Pseudo-retrosynthetic Tool for Synthetically Accessible *de novo* Design

Gian Marco Ghiandoni, Michael J. Bodkin, Beining Chen, Dimitar Hristozov, James E. A. Wallace, James Webster, and Valerie J. Gillet*© 2021 The Authors. Molecular Informatics published by Wiley-VCH GmbH. This is an open access article under the terms of the Creative Commons Attribution License, which permits use, distribution and reproduction in any medium, provided the original work is properly cited.

Supporting Information

1. Top 200 Drugs 2017 Retained Structures

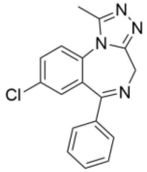	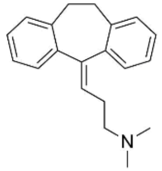	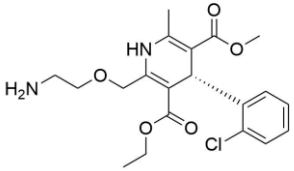	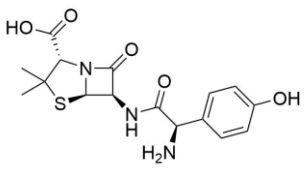	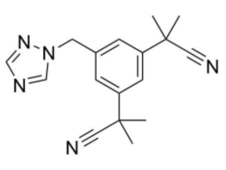
Alprazolam (*)	Amitriptyline	Amlodipine	Amoxicillin	Anastrozole
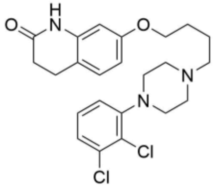	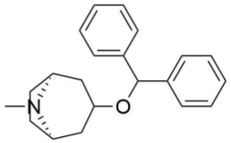	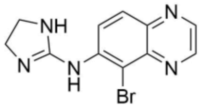	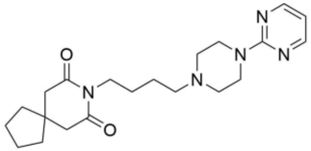	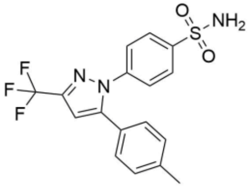
Aripiprazole	Benztropine	Brimonidine	Buspirone	Celecoxib
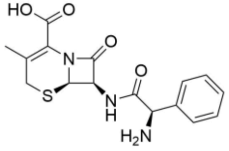	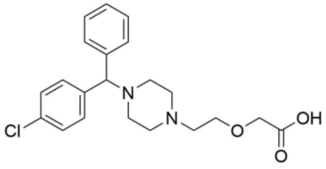	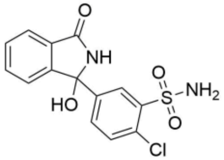	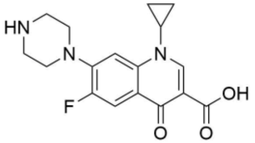	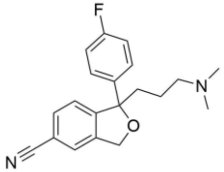
Cephalexin	Cetirizine	Chlorthalidone (*)	Ciprofloxacin	Citalopram (*)
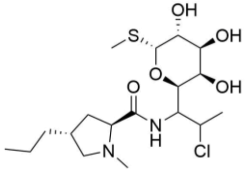	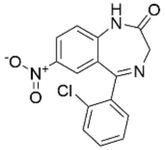	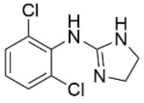	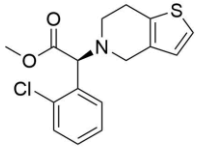	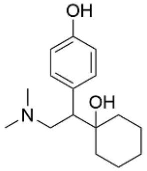
Clindamycin	Clonazepam (*)	Clonidine	Clopidogrel	Desvenlafaxine

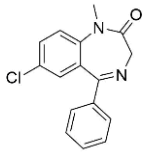	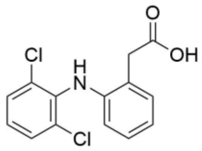	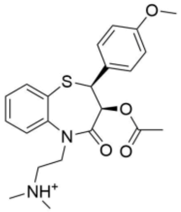	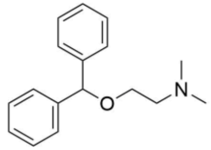	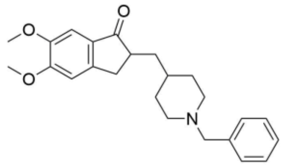
Diazepam (*)	Diclofenac	Diltiazem	Diphenhydramine (*)	Donepezil
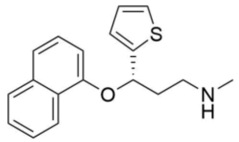	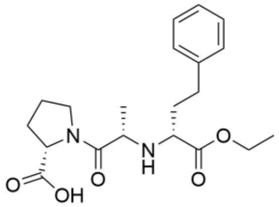	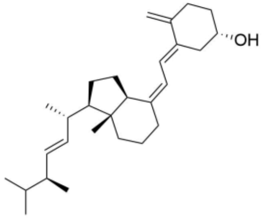	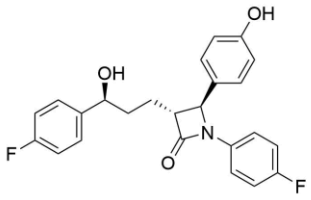	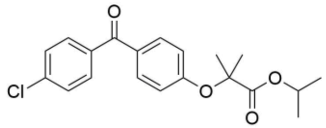
Duloxetine	Enalapril	Ergocalciferol	Ezetimibe	Fenofibrate
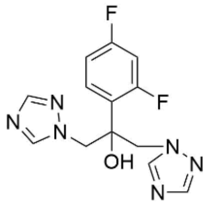	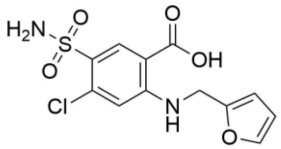	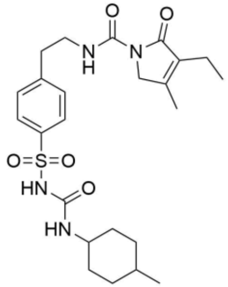	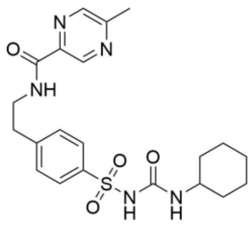	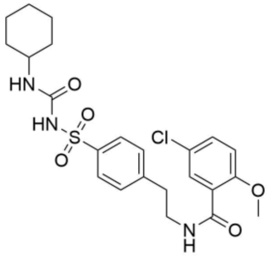
Fluconazole	Furosemide	Glimepiride	Glipizide	Glyburide
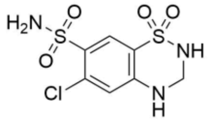	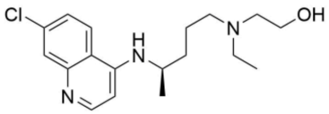	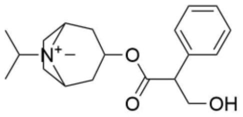	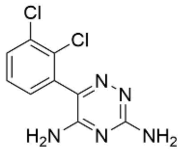	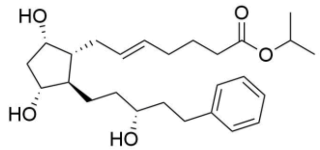
Hydrochlorothiazide (*)	Hydroxychloroquine	Ipratropium	Lamotrigine	Latanoprost

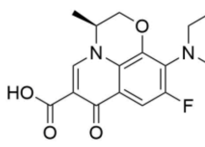	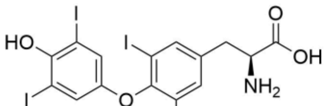	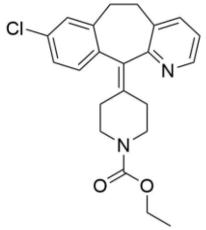	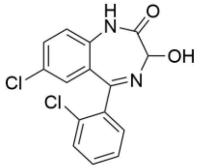	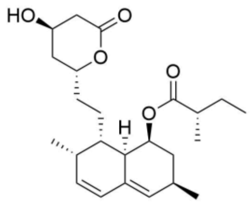
Levofloxacin	Levothyroxine	Loratadine	Lorazepam (*)	Lovastatin
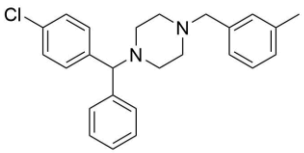	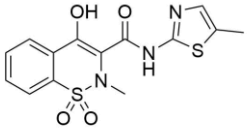	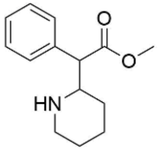	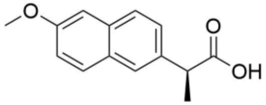	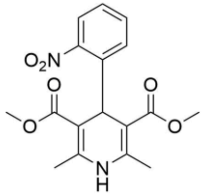
Meclizine	Meloxicam	Methylphenidate	Naproxen	Nifedipine
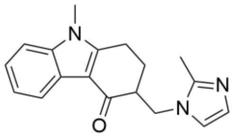	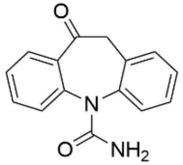	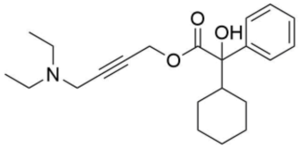	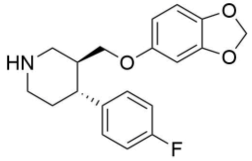	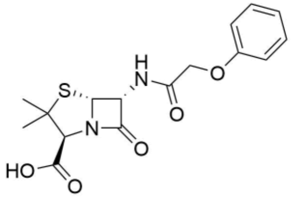
Ondansetron	Oxcarbazepine (*)	Oxybutynin	Paroxetine	Penicillin V
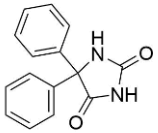	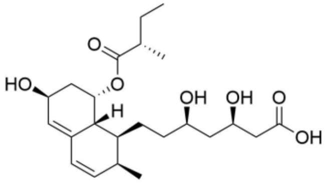	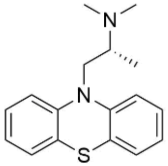	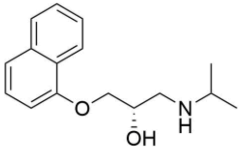	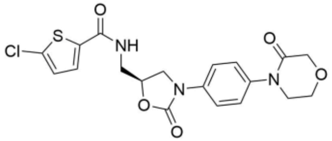
Phenytoin	Pravastatin	Promethazine	Propranolol	Rivaroxaban
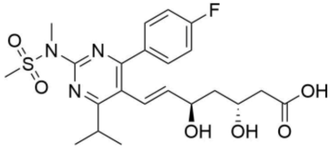	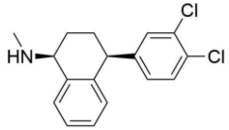	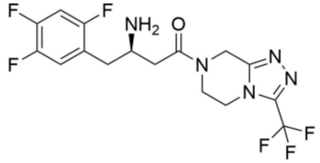	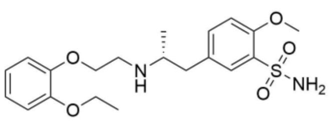	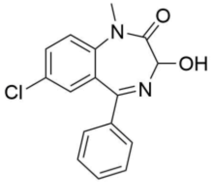
Rosuvastatin	Sertraline (*)	Sitagliptin	Tamsulosin	Temazepam (*)

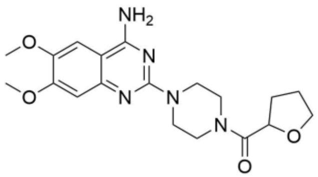	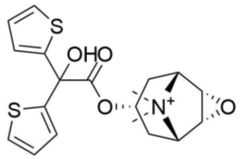	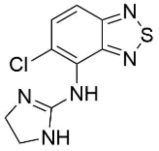	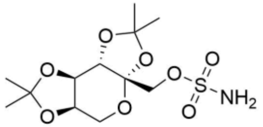	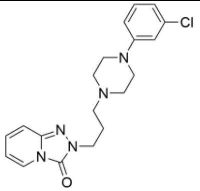
Terazosin	Tiotropium	Tizanidine	Topiramate	Trazodone
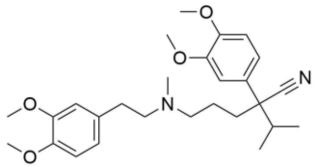	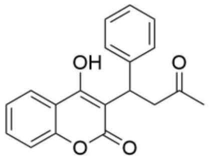	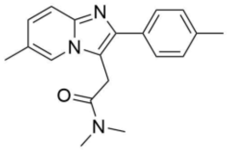		
Verapamil	Warfarin	Zolpidem		

Note: The structures that have failed the BRICS decomposition are marked by a star in brackets.

2. USPD and JMC design parameters used for the validation of RENATE

- minFragmentSize=1
- minKeyFragSize=5
- maxStartingMaterials=750
- maxReagents=1000
- queryHeavyAtomsAddThreshold=0.25
- numProductsCycle=4000
- numFinalProducts=1000
